# Crystal structure of 4-{[(cyano­imino)(methyl­sulfanyl)meth­yl]amino}-1,5-dimethyl-2-phenyl-2,3-di­hydro-1*H*-pyrazol-3-one

**DOI:** 10.1107/S2056989014027601

**Published:** 2015-01-01

**Authors:** Galal H. Elgemeie, Mamdouh Abouzeid, Peter G. Jones

**Affiliations:** a Chemistry Department, Faculty of Science, Helwan University, Cairo, Egypt; b Green Chemistry Department, National Research Center, Dokki, Cairo, Egypt; c Institut für Anorganische und Analytische Chemie, Technische Universität Braunschweig, Postfach 3329, D-38023 Braunschweig, Germany

**Keywords:** crystal structure, pyrazole, thio­carbamate, hydrogen bond

## Abstract

In the title compound, C_14_H_15_N_5_OS, the tautomer present in the solid state is that in which the immediately exocyclic N atom bears the H atom. The central five-membered ring is almost planar (r.m.s. deviation = 0.025 Å), but both its N atoms are significantly pyramidalized. A classical hydrogen bond from the N—H group to the cyanide N atom forms inversion-symmetric dimers, which are further linked by C—H⋯O inter­actions.

## Abstract 2

In the title compound, the tautomer present in the solid state is that in which the immediately exocyclic N atom bears the H atom. The central five-membered ring is planar, but both its N atoms are significantly pyramidalized.

## Chemical context   

The pyrazolone 4-amino-2,3-dimethyl-1-phenyl-3-pyrazolin-5-one (‘4-amino­anti­pyrine’) and its derivatives represent some of the most important compounds used as analgesic, anti­pyretic and anti-inflammatory drugs (Santos *et al.*, 2010 ▸). The biological activity of these compounds has been attributed to their scavenging activity against reactive oxygen and nitro­gen species in biochemical reactions (Costa *et al.*, 2006 ▸). Continu­ing our inter­est in the synthesis of azoles and of fused azoles as both potential CNS regulants and anti­metabolites in purine biochemical reactions (Elgemeie *et al.*, 1997 ▸, 2004*a* ▸,*b* ▸, 2005 ▸, 2007 ▸, 2008 ▸), our current work deals with the synthesis and structure of methyl *N*-cyano-*N*-imido­thio­carbamate derivatives of 4-amino­anti­pyrine derived from two-component reactions. The title compound (1) was synthesized by the condensation of 4-amino­anti­pyrine and *N*-cyano­imido-*S*,*S*-di­methyl­dithio­carbonate in a simple one-step reaction. Compound (1) can exist in two tautomeric forms: (1*a*) and (1*b*). The ^1^H and ^13^C NMR spectra cannot differentiate between the two structures. The X-ray structure determination was undertaken to establish the exact nature of the product.
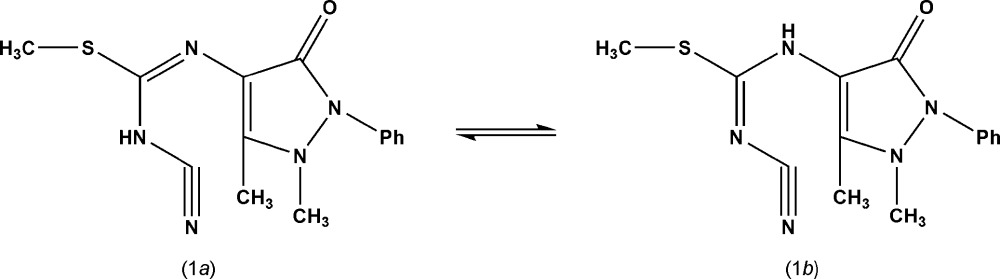


## Structural commentary   

**Figure 1:**
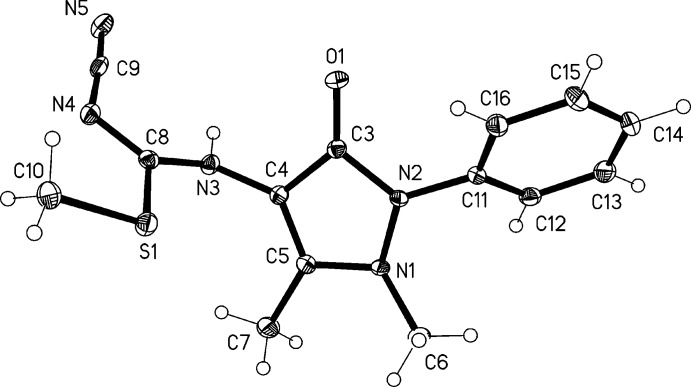
The mol­ecule of the title compound in the crystal, with displacement ellipsoids drawn at the 50% probability level.

The mol­ecule of (1) is shown in Fig. 1 ▸. The location and free refinement of the NH hydrogen atom confirm the existence of the tautomer (1*b*) in the solid state. The central five-membered ring is effectively planar (r.m.s. deviation 0.025 Å), but both its nitro­gen atoms are significantly pyramidalized, with C6 lying 0.635 (2) and C11 0.271 (2) Å out of the plane in opposite directions. The imido­thio­carbamate group is also roughly planar (r.m.s.d. 0.05 Å) and almost perpendicular to the central ring [inter­planar angle 83.38 (3)°]; the inter­planar phen­yl/di­hydro­pyrazole angle is 44.82 (5)°.

## Supra­molecular features   

**Figure 2:**
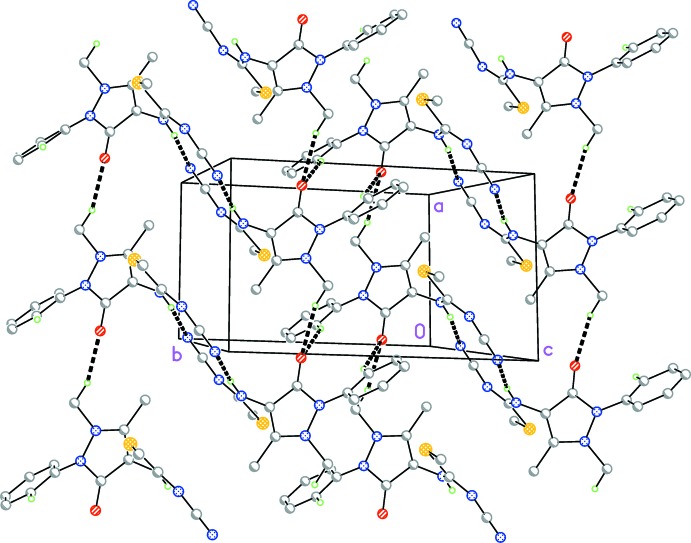
Packing diagram of the title compound. The view direction is rotated slightly from the vector perpendicular to the *ab* plane. Hydrogen bonds (one classical and two ‘weak’, the first three entries in Table 1 ▸) are drawn as thick dashed lines.

The main inter­molecular inter­action is the classical hydrogen bond from the NH function N3—H03 to the cyanide nitro­gen atom N5, forming inversion-symmetric dimers. These dimers are further linked in the *a*-axis direction by a pair of weak C—H⋯O hydrogen bonds to the same acceptor atom (C6—H6*C*⋯O1 and C16—H16⋯O1), forming a layer structure parallel to the *ab* plane (Fig. 2 ▸). See Table 1 ▸. The interaction C13—H13⋯N5 links the layers in the third dimension.

## Database survey   

The 1,5-dimethyl-2-phenyl-2,3-di­hydro-1*H*-pyrazol-3-one ring system with a nitro­gen substituent at the 4-position has been thoroughly investigated. A search of the Cambridge database (Version 5.35; Groom & Allen, 2014 ▸) gave 223 hits with 242 individual mol­ecules, mean bond lengths N1—N2 1.405, N2—C3 1.394, C3—C4 1.439, C4—C5 1.364, N1—C5 1.372 Å; all of these values agree closely with the bond lengths observed in the title compound.

## Synthesis and crystallization   

A solution of *N*-cyano­imido-*S*,*S*-di­methyl­dithio­carbonate (0.01 mol) in ethanol (20 ml) was added to a solution of 4-amino­anti­pyrine (0.01 mol) in ethanol (30 ml) containing catalytic amounts of piperidine (0.5 ml). The reaction mixture was heated at reflux for 30 min and then evaporated under reduced pressure. The yellow solid product was collected by filtration and recrystallized from ethanol, yield 85%, m.p. 489–491 K.

## Refinement   

Crystal data, data collection and structure refinement details are summarized in Table 2 ▸. The NH hydrogen atom was refined freely. Methyls were refined as idealized rigid groups that were allowed to rotate but not tip. Other H atoms were included using a riding model starting from calculated positions.

## Supplementary Material

Crystal structure: contains datablock(s) I, global. DOI: 10.1107/S2056989014027601/pk2542sup1.cif

Structure factors: contains datablock(s) I. DOI: 10.1107/S2056989014027601/pk2542Isup2.hkl

Supporting information file. DOI: 10.1107/S2056989014027601/pk2542Isup3.cml

CCDC reference: 1040102

Additional supporting information:  crystallographic information; 3D view; checkCIF report

## Figures and Tables

**Table 1:** Hydrogen-bond geometry (, )

*D*H*A*	*D*H	H*A*	*D* *A*	*D*H*A*
N3H03N5^i^	0.861(16)	2.125(16)	2.9386(13)	157.3(14)
C6H6*C*O1^ii^	0.98	2.30	3.2233(13)	156
C16H16O1^iii^	0.95	2.42	3.2318(13)	143
C13H13N5^iv^	0.95	2.57	3.3189(15)	136

Symmetry codes: (i) 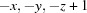; (ii) 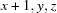; (iii) 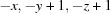; (iv) 
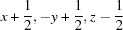
.

**Table 2:** Experimental details

Crystal data	
Chemical formula	C_14_H_15_N_5_OS
*M*_r_	301.37
Crystal system, space group	Monoclinic, *P*2_1_/*n*
Temperature (K)	100
*a*, *b*, *c* ()	7.3620(2), 11.9369(4), 16.6755(5)
()	100.191(3)
*V* (^3^)	1442.30(8)
*Z*	4
Radiation type	Mo *K*
(mm^1^)	0.23
Crystal size (mm)	0.40 0.35 0.12

Data collection	
Diffractometer	Oxford Diffraction Xcalibur Eos
Absorption correction	Multi-scan (*CrysAlis PRO*; Agilent, 2014 ▸)
*T*_min_, *T*_max_	0.913, 0.973
No. of measured, independent and observed [*I* > 2(*I*)] reflections	37668, 4359, 3829
*R*_int_	0.033
(sin /)_max_ (^1^)	0.724

Refinement	
*R*[*F* ^2^ > 2(*F* ^2^)], *wR*(*F* ^2^), *S*	0.033, 0.083, 1.08
No. of reflections	4359
No. of parameters	197
H-atom treatment	H atoms treated by a mixture of independent and constrained refinement
_max_, _min_ (e ^3^)	0.49, 0.28

Computer programs: *CrysAlis PRO* (Agilent, 2014 ▸), *SHELXS97*, *SHELXL97* and *XP* in *SHELXTL* (Sheldrick, 2008 ▸).

## supplementary crystallographic information

### Crystal data

**Table:**

C_14_H_15_N_5_OS	*F*(000) = 632
*M**_r_* = 301.37	*D*_x_ = 1.388 Mg m^−^^3^
Monoclinic, *P*2_1_/*n*	Mo *K*α radiation, λ = 0.71073 Å
Hall symbol: -P 2yn	Cell parameters from 11166 reflections
*a* = 7.3620 (2) Å	θ = 2.9–30.8°
*b* = 11.9369 (4) Å	µ = 0.23 mm^−^^1^
*c* = 16.6755 (5) Å	*T* = 100 K
β = 100.191 (3)°	Tablet, yellow
*V* = 1442.30 (8) Å^3^	0.40 × 0.35 × 0.12 mm
*Z* = 4	

### Data collection

**Table:**

Oxford Diffraction Xcalibur Eos diffractometer	4359 independent reflections
Radiation source: Enhance (Mo) X-ray Source	3829 reflections with *I* > 2σ(*I*)
Graphite monochromator	*R*_int_ = 0.033
Detector resolution: 16.1419 pixels mm^-1^	θ_max_ = 31.0°, θ_min_ = 2.5°
ω–scan	*h* = −10→10
Absorption correction: multi-scan (*CrysAlis PRO*; Agilent, 2014)	*k* = −16→17
*T*_min_ = 0.913, *T*_max_ = 0.973	*l* = −23→24
37668 measured reflections	

### Refinement

**Table:**

Refinement on *F*^2^	Primary atom site location: structure-invariant direct methods
Least-squares matrix: full	Secondary atom site location: difference Fourier map
*R*[*F*^2^ > 2σ(*F*^2^)] = 0.033	Hydrogen site location: inferred from neighbouring sites
*wR*(*F*^2^) = 0.083	H atoms treated by a mixture of independent and constrained refinement
*S* = 1.08	*w* = 1/[σ^2^(*F*_o_^2^) + (0.0355*P*)^2^ + 0.584*P*] where *P* = (*F*_o_^2^ + 2*F*_c_^2^)/3
4359 reflections	(Δ/σ)_max_ = 0.001
197 parameters	Δρ_max_ = 0.49 e Å^−^^3^
0 restraints	Δρ_min_ = −0.28 e Å^−^^3^

### Special details

**Table:**

Geometry. All e.s.d.'s (except the e.s.d. in the dihedral angle between two l.s. planes) are estimated using the full covariance matrix. The cell e.s.d.'s are taken into account individually in the estimation of e.s.d.'s in distances, angles and torsion angles; correlations between e.s.d.'s in cell parameters are only used when they are defined by crystal symmetry. An approximate (isotropic) treatment of cell e.s.d.'s is used for estimating e.s.d.'s involving l.s. planes.Least-squares planes (*x*,*y*,*z* in crystal coordinates) and deviations from them (* indicates atom used to define plane)5.7083 (0.0022) *x* + 6.9435 (0.0045) *y* + 1.7473 (0.0075) *z* = 5.2871 (0.0030)* 0.0075 (0.0007) C11 * -0.0046 (0.0008) C12 * -0.0024 (0.0008) C13 * 0.0067 (0.0008) C14 * -0.0038 (0.0008) C15 * -0.0032 (0.0008) C16Rms deviation of fitted atoms = 0.00501.8491 (0.0035) *x* + 6.5323 (0.0049) *y* + 12.3627 (0.0056) *z* = 8.2341 (0.0008)Angle to previous plane (with approximate e.s.d.) = 44.82 (0.05)* -0.0339 (0.0006) N1 * 0.0321 (0.0006) N2 * -0.0180 (0.0006) C3 * -0.0029 (0.0006) C4 * 0.0226 (0.0006) C5 0.6346 (0.0017) C6 - 0.2707 (0.0016) C11 - 0.0906 (0.0015) O1 - 0.1072 (0.0016) N3 0.0915 (0.0018) C7Rms deviation of fitted atoms = 0.02465.0497 (0.0013) *x* - 8.5455 (0.0018) *y* + 0.1177 (0.0066) *z* = 0.2502 (0.0039)Angle to previous plane (with approximate e.s.d.) = 83.38 (0.03)* -0.0121 (0.0005) N3 * 0.0218 (0.0008) C8 * 0.1054 (0.0008) N4 * 0.0159 (0.0008) C9 * -0.0687 (0.0007) N5 * -0.0211 (0.0005) S1 * -0.0412 (0.0005) C10Rms deviation of fitted atoms = 0.0519
Refinement. Refinement of *F*^2^ against ALL reflections. The weighted *R*-factor *wR* and goodness of fit *S* are based on *F*^2^, conventional *R*-factors *R* are based on *F*, with *F* set to zero for negative *F*^2^. The threshold expression of *F*^2^ > σ(*F*^2^) is used only for calculating *R*-factors(gt) *etc*. and is not relevant to the choice of reflections for refinement. *R*-factors based on *F*^2^ are statistically about twice as large as those based on *F*, and *R*- factors based on ALL data will be even larger.The NH hydrogen was refined freely. Methyls were refined as idealized rigid groups allowed to rotate but not tip. Other H were included using a riding model starting from calculated positions.

### Fractional atomic coordinates and isotropic or equivalent isotropic displacement parameters (Å^2^)

**Table:**

	*x*	*y*	*z*	*U*_iso_*/*U*_eq_	
S1	0.46361 (4)	0.25628 (2)	0.663545 (16)	0.01610 (7)	
O1	0.05702 (10)	0.34546 (7)	0.46766 (5)	0.01656 (16)	
N1	0.48581 (11)	0.34600 (7)	0.40783 (5)	0.01156 (16)	
N2	0.31507 (11)	0.39592 (7)	0.41232 (5)	0.01173 (16)	
N3	0.28997 (12)	0.15084 (7)	0.53431 (5)	0.01269 (17)	
H03	0.205 (2)	0.1073 (13)	0.5091 (9)	0.025 (4)*	
N4	0.21528 (12)	0.09469 (7)	0.66041 (5)	0.01476 (17)	
N5	−0.03210 (14)	−0.03206 (8)	0.59200 (6)	0.01949 (19)	
C3	0.21686 (13)	0.32907 (8)	0.45828 (6)	0.01160 (18)	
C4	0.34313 (14)	0.24011 (8)	0.48763 (6)	0.01152 (18)	
C5	0.50320 (14)	0.25429 (8)	0.45825 (6)	0.01172 (18)	
C6	0.63812 (14)	0.42144 (9)	0.39925 (7)	0.0161 (2)	
H6A	0.6430	0.4829	0.4385	0.024*	
H6B	0.6186	0.4520	0.3438	0.024*	
H6C	0.7547	0.3799	0.4097	0.024*	
C7	0.67468 (15)	0.18602 (9)	0.47425 (7)	0.0190 (2)	
H7A	0.6539	0.1193	0.5056	0.029*	
H7B	0.7749	0.2306	0.5053	0.029*	
H7C	0.7083	0.1632	0.4224	0.029*	
C8	0.30606 (14)	0.15750 (8)	0.61605 (6)	0.01240 (18)	
C9	0.08493 (14)	0.02760 (9)	0.62107 (6)	0.01450 (19)	
C10	0.43222 (16)	0.24152 (10)	0.76777 (6)	0.0194 (2)	
H10A	0.3071	0.2651	0.7726	0.029*	
H10B	0.5223	0.2884	0.8030	0.029*	
H10C	0.4502	0.1630	0.7845	0.029*	
C11	0.23608 (13)	0.47889 (8)	0.35581 (6)	0.01204 (18)	
C12	0.26102 (14)	0.47700 (9)	0.27495 (6)	0.0150 (2)	
H12	0.3340	0.4203	0.2563	0.018*	
C13	0.17747 (15)	0.55933 (10)	0.22197 (7)	0.0192 (2)	
H13	0.1938	0.5591	0.1667	0.023*	
C14	0.07053 (16)	0.64172 (10)	0.24912 (7)	0.0215 (2)	
H14	0.0152	0.6983	0.2127	0.026*	
C15	0.04415 (15)	0.64168 (10)	0.32949 (7)	0.0197 (2)	
H15	−0.0308	0.6976	0.3477	0.024*	
C16	0.12666 (14)	0.56035 (9)	0.38346 (6)	0.0153 (2)	
H16	0.1087	0.5603	0.4385	0.018*	

### Atomic displacement parameters (Å^2^)

**Table:**

	*U*^11^	*U*^22^	*U*^33^	*U*^12^	*U*^13^	*U*^23^
S1	0.01678 (13)	0.01752 (13)	0.01357 (12)	−0.00532 (9)	0.00148 (9)	0.00014 (9)
O1	0.0108 (3)	0.0214 (4)	0.0189 (4)	0.0022 (3)	0.0065 (3)	0.0028 (3)
N1	0.0086 (4)	0.0125 (4)	0.0142 (4)	0.0021 (3)	0.0038 (3)	0.0015 (3)
N2	0.0089 (4)	0.0146 (4)	0.0126 (4)	0.0034 (3)	0.0042 (3)	0.0026 (3)
N3	0.0138 (4)	0.0112 (4)	0.0131 (4)	−0.0028 (3)	0.0023 (3)	0.0005 (3)
N4	0.0149 (4)	0.0149 (4)	0.0147 (4)	−0.0015 (3)	0.0033 (3)	0.0018 (3)
N5	0.0216 (5)	0.0204 (5)	0.0179 (4)	−0.0058 (4)	0.0072 (4)	0.0003 (3)
C3	0.0116 (4)	0.0131 (4)	0.0102 (4)	−0.0005 (3)	0.0024 (3)	−0.0003 (3)
C4	0.0119 (4)	0.0112 (4)	0.0116 (4)	−0.0002 (3)	0.0024 (3)	0.0003 (3)
C5	0.0115 (4)	0.0114 (4)	0.0123 (4)	0.0010 (3)	0.0019 (3)	−0.0008 (3)
C6	0.0107 (4)	0.0164 (5)	0.0213 (5)	−0.0020 (4)	0.0029 (4)	0.0030 (4)
C7	0.0140 (5)	0.0173 (5)	0.0263 (5)	0.0054 (4)	0.0052 (4)	0.0054 (4)
C8	0.0112 (4)	0.0111 (4)	0.0148 (4)	0.0017 (3)	0.0021 (3)	0.0009 (3)
C9	0.0168 (5)	0.0143 (4)	0.0141 (4)	0.0009 (4)	0.0072 (4)	0.0028 (3)
C10	0.0200 (5)	0.0251 (6)	0.0125 (5)	−0.0010 (4)	0.0015 (4)	−0.0013 (4)
C11	0.0099 (4)	0.0133 (4)	0.0127 (4)	0.0003 (3)	0.0014 (3)	0.0021 (3)
C12	0.0119 (4)	0.0197 (5)	0.0140 (4)	0.0006 (4)	0.0038 (4)	0.0008 (4)
C13	0.0156 (5)	0.0285 (6)	0.0137 (5)	0.0001 (4)	0.0027 (4)	0.0060 (4)
C14	0.0164 (5)	0.0252 (6)	0.0226 (5)	0.0040 (4)	0.0024 (4)	0.0118 (4)
C15	0.0171 (5)	0.0181 (5)	0.0245 (6)	0.0067 (4)	0.0051 (4)	0.0047 (4)
C16	0.0149 (5)	0.0165 (5)	0.0151 (5)	0.0032 (4)	0.0043 (4)	0.0014 (4)

### Geometric parameters (Å, º)

**Table:**

S1—C8	1.7430 (10)	C13—C14	1.3850 (17)
S1—C10	1.8021 (11)	C14—C15	1.3878 (16)
O1—C3	1.2298 (12)	C15—C16	1.3884 (14)
N1—C5	1.3725 (13)	N3—H03	0.861 (16)
N1—N2	1.4051 (11)	C6—H6A	0.9800
N1—C6	1.4647 (13)	C6—H6B	0.9800
N2—C3	1.3938 (12)	C6—H6C	0.9800
N2—C11	1.4189 (12)	C7—H7A	0.9800
N3—C8	1.3495 (13)	C7—H7B	0.9800
N3—C4	1.4150 (13)	C7—H7C	0.9800
N4—C8	1.3157 (13)	C10—H10A	0.9800
N4—C9	1.3303 (14)	C10—H10B	0.9800
N5—C9	1.1562 (14)	C10—H10C	0.9800
C3—C4	1.4389 (14)	C12—H12	0.9500
C4—C5	1.3644 (14)	C13—H13	0.9500
C5—C7	1.4866 (14)	C14—H14	0.9500
C11—C16	1.3923 (14)	C15—H15	0.9500
C11—C12	1.3928 (14)	C16—H16	0.9500
C12—C13	1.3901 (15)		
			
C8—S1—C10	100.58 (5)	C8—N3—H03	117.0 (10)
C5—N1—N2	107.03 (8)	C4—N3—H03	115.6 (10)
C5—N1—C6	124.07 (8)	N1—C6—H6A	109.5
N2—N1—C6	116.87 (8)	N1—C6—H6B	109.5
C3—N2—N1	110.03 (8)	H6A—C6—H6B	109.5
C3—N2—C11	124.98 (8)	N1—C6—H6C	109.5
N1—N2—C11	121.73 (8)	H6A—C6—H6C	109.5
C8—N3—C4	121.86 (9)	H6B—C6—H6C	109.5
C8—N4—C9	117.37 (9)	C5—C7—H7A	109.5
O1—C3—N2	125.45 (9)	C5—C7—H7B	109.5
O1—C3—C4	130.48 (9)	H7A—C7—H7B	109.5
N2—C3—C4	104.05 (8)	C5—C7—H7C	109.5
C5—C4—N3	129.16 (9)	H7A—C7—H7C	109.5
C5—C4—C3	109.47 (9)	H7B—C7—H7C	109.5
N3—C4—C3	121.20 (9)	S1—C10—H10A	109.5
C4—C5—N1	109.03 (9)	S1—C10—H10B	109.5
C4—C5—C7	128.87 (9)	H10A—C10—H10B	109.5
N1—C5—C7	122.10 (9)	S1—C10—H10C	109.5
N4—C8—N3	124.87 (9)	H10A—C10—H10C	109.5
N4—C8—S1	119.57 (8)	H10B—C10—H10C	109.5
N3—C8—S1	115.55 (8)	C13—C12—H12	120.5
N5—C9—N4	175.27 (11)	C11—C12—H12	120.5
C16—C11—C12	121.00 (9)	C14—C13—H13	119.8
C16—C11—N2	117.43 (9)	C12—C13—H13	119.8
C12—C11—N2	121.53 (9)	C13—C14—H14	120.0
C13—C12—C11	118.98 (10)	C15—C14—H14	120.0
C14—C13—C12	120.50 (10)	C14—C15—H15	119.8
C13—C14—C15	120.01 (10)	C16—C15—H15	119.8
C14—C15—C16	120.42 (10)	C15—C16—H16	120.5
C15—C16—C11	119.08 (10)	C11—C16—H16	120.5
			
C5—N1—N2—C3	−6.47 (11)	N2—N1—C5—C7	−174.80 (9)
C6—N1—N2—C3	−150.77 (9)	C6—N1—C5—C7	−33.74 (15)
C5—N1—N2—C11	−167.02 (9)	C9—N4—C8—N3	−7.03 (15)
C6—N1—N2—C11	48.68 (12)	C9—N4—C8—S1	174.19 (8)
N1—N2—C3—O1	−173.69 (9)	C4—N3—C8—N4	159.83 (10)
C11—N2—C3—O1	−13.92 (16)	C4—N3—C8—S1	−21.35 (13)
N1—N2—C3—C4	4.76 (10)	C10—S1—C8—N4	−3.77 (9)
C11—N2—C3—C4	164.53 (9)	C10—S1—C8—N3	177.34 (8)
C8—N3—C4—C5	98.29 (13)	C3—N2—C11—C16	53.45 (14)
C8—N3—C4—C3	−87.05 (12)	N1—N2—C11—C16	−149.00 (9)
O1—C3—C4—C5	176.99 (11)	C3—N2—C11—C12	−124.26 (11)
N2—C3—C4—C5	−1.35 (11)	N1—N2—C11—C12	33.28 (14)
O1—C3—C4—N3	1.38 (17)	C16—C11—C12—C13	1.19 (16)
N2—C3—C4—N3	−176.97 (9)	N2—C11—C12—C13	178.82 (10)
N3—C4—C5—N1	172.55 (9)	C11—C12—C13—C14	−0.23 (17)
C3—C4—C5—N1	−2.60 (11)	C12—C13—C14—C15	−0.84 (18)
N3—C4—C5—C7	−7.13 (18)	C13—C14—C15—C16	0.97 (18)
C3—C4—C5—C7	177.71 (10)	C14—C15—C16—C11	−0.03 (17)
N2—N1—C5—C4	5.49 (11)	C12—C11—C16—C15	−1.06 (16)
C6—N1—C5—C4	146.55 (9)	N2—C11—C16—C15	−178.78 (10)

### Hydrogen-bond geometry (Å, º)

**Table:**

*D*—H···*A*	*D*—H	H···*A*	*D*···*A*	*D*—H···*A*
N3—H03···N5^i^	0.861 (16)	2.125 (16)	2.9386 (13)	157.3 (14)
C6—H6*C*···O1^ii^	0.98	2.30	3.2233 (13)	156
C16—H16···O1^iii^	0.95	2.42	3.2318 (13)	143
C13—H13···N5^iv^	0.95	2.57	3.3189 (15)	136

Symmetry codes: (i) −*x*, −*y*, −*z*+1; (ii) *x*+1, *y*, *z*; (iii) −*x*, −*y*+1, −*z*+1; (iv) *x*+1/2, −*y*+1/2, *z*−1/2.
